# Assessing the exploitation status of *Johnius belangerii* in Zhanjiang Bay

**DOI:** 10.1371/journal.pone.0314230

**Published:** 2024-11-21

**Authors:** Siman Deng, Dongrong Liao, Kun Lin, Shaoliang Lyu, Ning Chen, Xuefeng Wang

**Affiliations:** 1 Fisheries College, Guangdong Ocean University, Zhanjiang, Guangdong Province, China; 2 CAS Key Laboratory of Tropical Marine Bio-resources and Ecology, Guangdong Provincial Key Laboratory of Applied Marine Biology, South China Sea Institute of Oceanology, Chinese Academy of Sciences, Guangzhou, China; 3 University of Chinese Academy of Sciences, Beijing, China; CIFRI: Central Inland Fisheries Research Institute, INDIA

## Abstract

Fishery stock assessment is the basis of fishery management. This study explored the applicability of the Length-Based Spawning Potential Ratio method (LBSPR) to assess the exploitation status of data-limited fisheries. Using data from bottom trawl surveys in Zhanjiang Bay, the study estimated the relative fishing mortality (*F/M*) of *Johnius belangerii* stock. The results showed that the average lengths at 50% and 95% selectivity (*L*_S50_ and *L*_S95_) were 105 mm and 145 mm, respectively, both of which are smaller than the lengths at 50% and 95% sexual maturity length (*L*_50_ = 125 mm and *L*_95_ = 167 mm) determined via the logistic curve. The estimated spawning potential ratio (SPR) was 0.15 significantly below the reference threshold of 0.2. The study recommended the establishment of a minimum size range (137 to 150 mm) length for harvesting *Johnius belangerii* to enhance their reproductive potential. It also emphasized the importance of scientific monitoring of fishery resources and the ecological environment in Zhanjiang Bay to ensure sustainable management.

## 1. Introduction

Fishery resources are a crucial component of the fishery economy, providing abundant animal proteins for humans and generating significant ecological and environmental benefits [1]. Assessing fishery resources is essential for developing sustainable fishery management strategies, with biological reference points from these assessments serving as a foundation for management decisions. Globally, the majority of fisheries are data-limited [2], with over 90% of species or stocks experiencing issues such as lack of long-term stock surveys, data discontinuity and incomplete surveys and statistics [3]. Traditional stock assessment methods, which require precise biological data and complete catch records, are often unsuitable for these fisheries. In 2013, the World Conference on Stock Assessment Methods (WCAM) identified "data-limited methods"(DLM) as a key theme, highlighting the need for resource assessment methods that accommodate data-limited situations [4]. Research on DLM has since become a focal point in fishery resource studies, particularly in regions where data limitations are prevalent [5].

Length data, which can be easily collected from fishery surveys [6], Are often used in fishery stock assessment due to the correlation between age and length. Length data can be converted to age using growth equation, enabling resource assessments based on age structure. Consequently, researchers frequently employ length frequency data as the basic for these length [7–9]. Asymptotic length is an important parameter in basic biological research, reflecting the growth characteristics and changes, and is used to estimate exploitation parameters and predict catches [10]. Length-based methods have been widely adopted to estimate biological parameters and understand the dynamics of marine populations [11].

Spawning Potential Ratio (SPR) is a recognized biological reference point. The International Council for the Exploration of the Sea (ICES) has stated in relevant workshops that Length-Based Indices (LBI) and Length-Based Spawning Potential Ratio (LBSPR) are the most appropriate methods to estimate SPR for achieving reliable stock assessments [12]. Compared to the LBI, LBSPR requires simpler model inputs and does not necessitate annul catch/landings length composition data, providing metrics that more accurately reflect stock conditions [13]. The spawning potential ratio (SPR) measures the proportion of reproductive potential unharvested under various fishing pressures, ranging from 100% in unexploited stocks to 0% in stocks that do not spawn. LBSPR method, developed for data-limited fisheries, uses a maximum likelihood approach to fit length frequency data by inputting parameters such as the *M/k* ratio, *L*_∞_, and knowledge of maturity (L_*50*_ and L_*95*_, which are lengths at 50% and 95% maturity, respectively) distribution curves and expected growth curves to estimate SPR values. This method commonly used to study the resourcefulness and reproductive supplementation characteristics of populations in the present situation of limited survey data [14]. Ernawati et al. analyzed the reproductive potential of *Malabaricus perch* in the western part of South Sulawesi using the LBSPR method and taking into account uncertainties [15]. Cousido-Rocha et al. applied the LBI and LBSPR methods to analyze the population status of fishery resources in the Bay of Biscay and the Iberian Coast ecoregion and analyzed the parameter sensitivities of the two methods [13]. All these studies used LBSPR to assess the current population status of a fish species. Additionally, they have utilized readily available data to simulate the resource status, thereby facilitating more timely interventions for management measures. Since 2014, the LBSPR model has been extensively validated and increasingly applied in marine resource assessments [16–19].

In Zhanjiang Bay, the Belanger’s croaker, *Johnius belangerii* (Sciaenidae, Perciformes) is a dominant species in the fishery resources Zhanjiang Bay [20]. but suffers from incomplete and discontinuous long-term monitoring, classifying it as a data-limited fishery. *Johnius belangerii* plays a significant role in the marine food web further in marine ecosystem [21]. The species is distributed across the Yellow Sea, Bohai Sea, and northern South China Sea [22]. Despite numerous studies on its feeding and growth characteristics in various regions [23–25], there has been no specific research on the stock status of *Johnius belangerii* in Zhanjiang Bay. Yang Jiming et al. [23] revealed the dietary characteristics of *Johnius belangerii* in the Bohai Sea through intragastric food analysis. Xue Ying et al. [24] used extensive datasets and multivariate statistical analyses to compare the dietary characteristics of three otoliths species in the South Yellow Sea. Li Zhongyi et al. [25] analyzed the dietary composition of *Johnius belangerii* in the Yangtze River estuary and the South Yellow Sea, as well as the comparison among the small yellow croaker in autumn, using stable isotope analyses. Numerous articles have investigated the biological characteristics of *Johnius belangerii* in various marine environments, including but not limited to the Pearl River Estuary, Haizhou Bay, Xiamen Sea and Liusha Bay.

This study aims to assess the exploitation status of *Johnius belangerii* in Zhanjiang Bay using LBSPR method, calculating SPR values and other biological reference points, to inform conservation strategy. The goal is to provide a robust reference for the stock assessment and scientific management of the nearshore pelagic fishes in the northern South China Sea.

## 2. Materials and methods

### 2.1. Data collection

**Fig 1:**
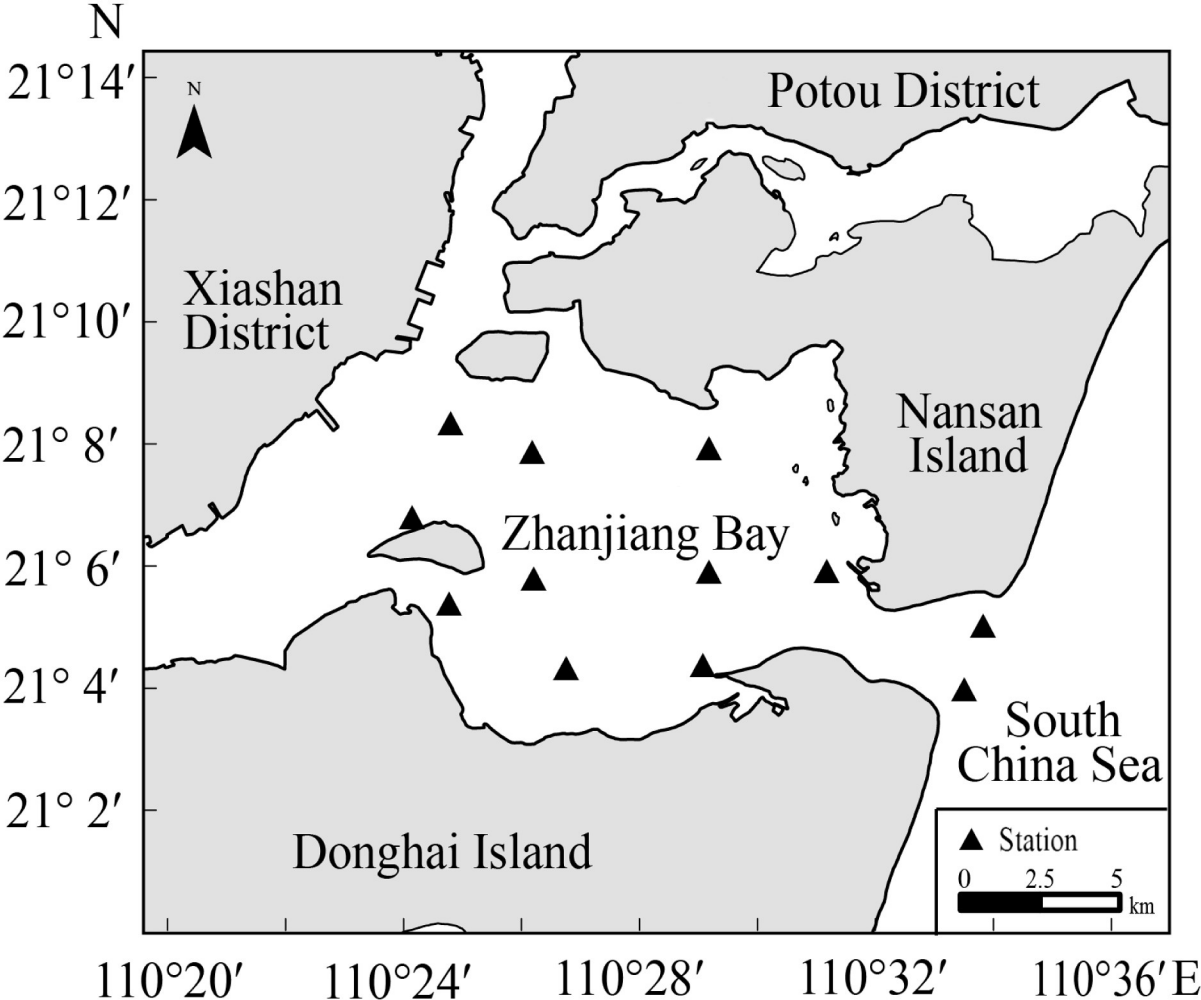
The survey station in Zhanjiang Bay in 2020. Source: Liao D R, Li A X, Chen N, Lyu S L, Wang X F. Biological characteristics and resource exploitation status of *Johnius belangerii* population in Zhanjiang Bay[J]. South China Fisheries Science, 2024, 20(3):27.

The study based on the data from the 2020 bottom trawl survey in Zhanjiang Bay (Fig 1). The survey station was strategically established considering prevailing fishing dynamics and oceanic variables within the harbour. The survey was conducted during the following months: April (spring), August (summer), October and November (autumn), as well as January and December (winter). During the summer breeding season, *Johnius belangerii* are more abundant, making collection more challenging. A total of 842 samples of *Johnius belangerii* were collected, with 262 sampled in spring, 84 in summer, 208 in autumn and 288 in winter. Detailed biological measurements were carried out 500 individuals, with 146 sampled in the spring, 74 in the summer, 74 in the autumn, and 206 in the winter. The remaining specimens were measured for length and weight only (Table 1). The survey vessel measured 16.7 meters in length and 4.0 meters in width, with a main engine power of 79 kW. The bottom trawl net had a mesh width of 11 meters, a mesh length of 40 mm, and a codend mesh length of 25 mm. The survey adhered to the "Specification for Marine Fisheries Resources Investigation" (SC/T 9403–2012) guidelines [26].

**Table 1:** Relevant data collection of *Johnius belangerii*.

	Total scope	Average value	Range of strengths and percentage
Length (mm)	27–174	109±22	91–130 (63.42%)
Weight (g)	0.33–125.22	29.80±18.15	10–35 (58.08%)

Comprehensive biological measurements and subsequent analyses were meticulously conducted. These measurements included length, weight, and gonadal maturity. The relationship between length and weight was modeled using the power function. Analysis of sex ratio and gonadal development analysis provided significant insights into maturity stages. Biological measurements of *Johnius belangerii* included length, weight, and gonadal maturity, with accuracies of length1mm and 0.1 g, respectively.

Using the frequency distribution method, the length and weight of *Johnius belangerii* were grouped at intervals of 10 mm and 5 g, respectively. Length groups accounting for more than 10% of the total sample size were identified as the dominant length and weight groups.

### 2.2. Parameter calculation

The relationship between length and weight in fish was described by a power function [27]:

$$W=aL^{b}$$ (1)

where *W* represents weight (g); *L* represents the length (mm); *a* and *b* are the parameters. Specifically, *a* encapsulates environmental conditions impacting habitat quality, while *b* delineates the allometric growth factor, reflecting the non-uniform growth and development of the fish.

For sex ratio and gonadal maturity analysis, individuals were categorized into groups at 10 mm intervals of length, allowing for the computation of male and female proportions within each bracket. The chi-square test was used to determine the disparity between observed male-female ratios and the expected 1:1 ratio. Gonadal maturity was categorized into stages I-VI using West’s method, with individual stage III or beyond as sexually mature [28].

Life history parameters, such as the initial sexual maturity length (*L*_50_) and 95% sexual maturity length (*L*_95_) of *Johnius belangerii*, were derived through logistic curve fitting in R software using the formula:

$$P_{i}=1/[1+e^{-(c+dl_{i})}]$$ (2)

where *P*_*i*_ represents the percentage of mature individuals within each length group; *L*_*i*_ represents the median value of each length group (mm), and *c*and *d* are the estimated parameters.

The asymptotic length (*L*_∞_) and growth coefficient (*k*) of *Johnius belangerii* were estimated using ELEFAN I method within FISAT II software. Subsequently, the total mortality (*Z*) was calculated using the length-converted catch curve approach. To ensure the accuracy of these estimates, the life history parameters were re-evaluated using TropicFishR Package as an independent validation of the initial method.

Natural mortality (*M*) was estimated by Paul’s formular [29]:

$$\ln\;M=-0.0066-0.279\;\ln\;L_{\infty }+0.6543lnk+0.4634\;\ln\;T$$ (3)

where *M* denotes natural mortality; *L*_∞_ is the asymptotic length; *k* is the growth coefficient, and *T* is the annual average temperature at the survey area in Zhanjiang Bay.

Fishing mortality (*F*) was calculated as the difference between total mortality (*Z*) and natural mortality:

F=Z−M (4)

The exploitation rate (*E*), a key indicator of stock status, is determined by the proportion of fishing mortality to total mortality:

E=F/Z (5)

The *M*/*k* represents the ratio of the natural mortality to the growth parameter *k* in the Von Bertalanffy equation.

### 2.3. Evaluation method

The Spawning Potential Ratio (SPR) delineates the fraction of unexploited reproductive capability under specific fishing pressures and serves as a pivotal determinant for establishing fishing limitations. For an untouched population, the SPR is 100%, while populations devoid of spawning due to factors such as the removal of all mature fish or capture of all female fish have an SPR of zero.

The LBSPR method relies on a population dynamics model assuming equal capture capabilities between female and male fish [14]. Based on an improved age structure model, annual data is converted into monthly data according to actual conditions, with natural mortality is assumed as a constant. Von Bertalanffy growth function was used to describe the population growth. Adjustments to the probability matrix were made according to anticipated age-length distributions within the catch. Sequential computations allowed for the depiction of length selectivity, crucial in conjunction with *Z/k* interactions, to ascertain the length composition of the catch. The SPR is calculated through parameter substitution into the following formula:

$$SPR=\frac{\sum {\left(1-{\tilde{L}}_{x}\right)}^{\left(M/k[(F/M)+1]\right)}{\tilde{L}}_{x}^{b}}{\sum {1-{\tilde{L}}_{x}}^{M/k}{\tilde{L}}_{x}^{b}}\;for\;x_{m}\leq x\leq 1$$ (6)

where *L*_x_ is the length; *M* is the natural mortality; *k* is the growth parameter; *F* is the fishing mortality, and *b* is a power exponent approximating 3.

The derived SPR value was compared against the target reference point of 0.2 to evaluate the current exploitation status of *Johnius belangerii* in Zhanjiang Bay.

### 2.4. Evaluation indicator

Studies have shown that *Z*⁄*k*≤3 indicates that natural mortality is the main cause of fish mortality, whereas *Z*⁄*k*>3 indicates that fishing is the primary cause [27]. According to Shijie Zhou et al. [30], the relationship between the fishing mortality coefficient (*F*_*MSY*_) corresponding to the maximum sustainable yield of *Osteichthyes* and the natural mortality coefficient *M* is *F*_*MSY*_ = 0.87*M*, i.e., the relative fishing mortality rate corresponding to the maximum sustainable yield is *F*_*MSY*_/*M* = 0.87.

### 2.5. Evaluation model

**Fig 2:**
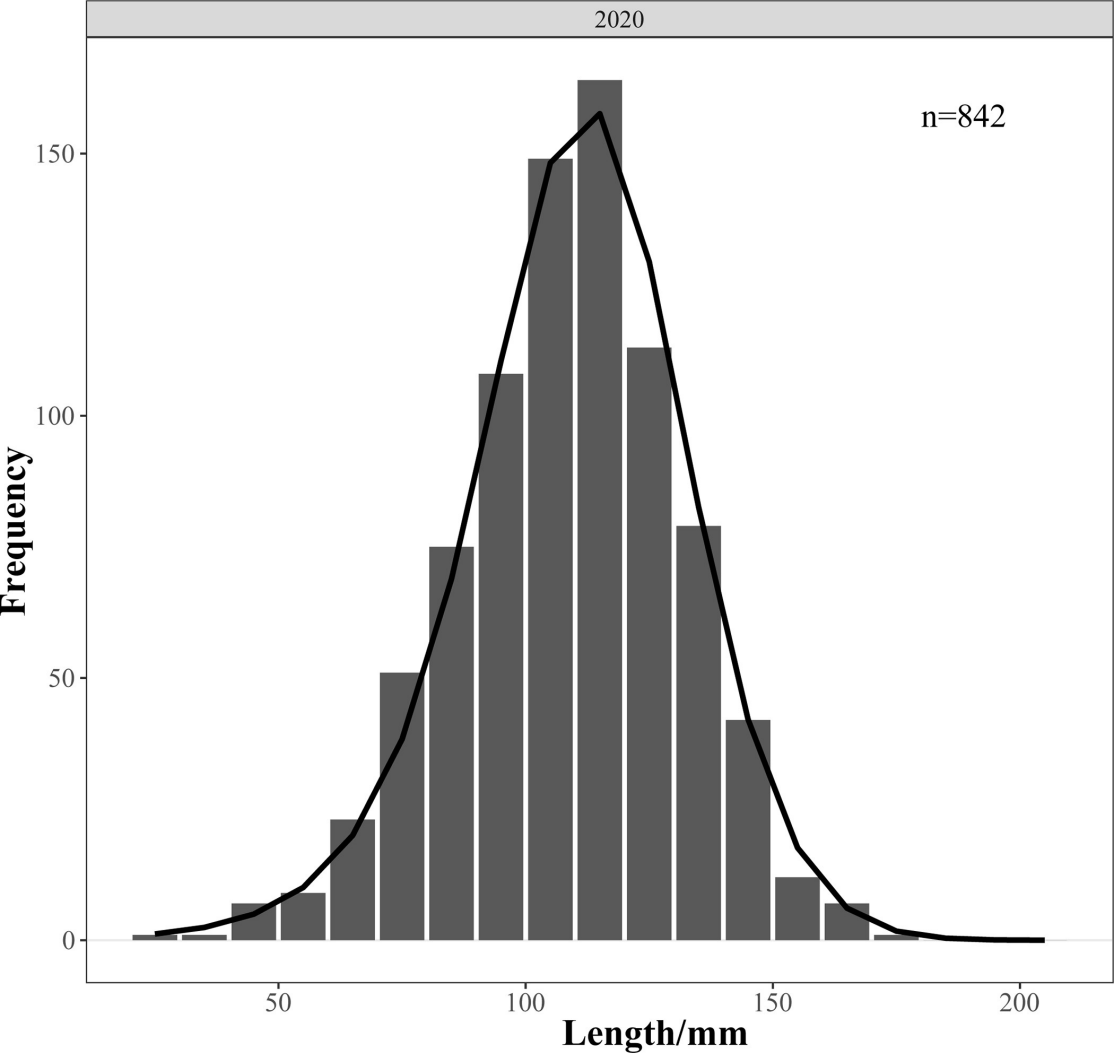
Length frequency distribution of *J*.*belangerii* in Zhanjiang Bay and model fitting curves.

**Fig 3:**
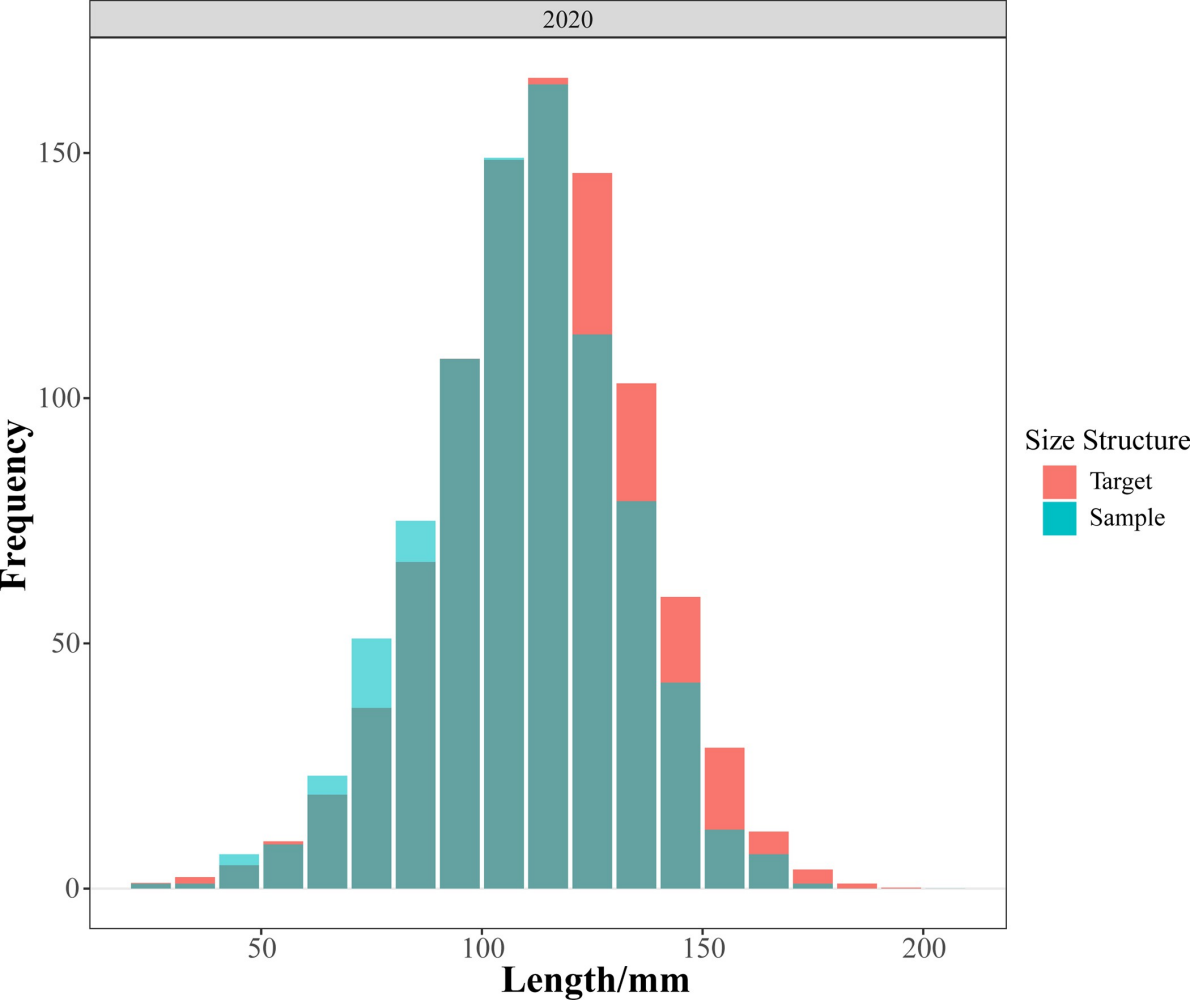
Comparison of expected length structure and sample length structure.

The length frequency distribution curve obtained by fitting the LBSPR model conformed to a normal distribution and matched the trend of actual length composition data, indicating the fitting is applicable (Fig 2). Simulation results showed that, under the existing gear selectivity in Zhanjiang Bay, the length distribution of samples for *Johnius belangerii* in 2020 was smaller compared to the expected distribution in an unfished state, with a scarcity of larger individuals, indicating a phenomenon of individual miniaturization (Fig 3).

## 3 Results

The length of *Johnius belangerii* ranged from 27 to 174 mm, with an average length of 109 ± 22 mm. The dominant length group was 91 to 130 mm comprising 63.42% of the samples. The weight ranged from 0.33 to 125.22 g, with an average weight of 29.80 ± 18.15 g and a dominant weight of 10 to 35 g, accounting for 58.08% of the samples. In total, 842 samples of *Johnius belangerii* were collected in Zhanjiang Bay in 2020, including 288 in winter, 262 in spring, 84 in summer, and 208 in autumn.

### 3.1. Seasonal variations of length and weight distribution

**Fig 4:**
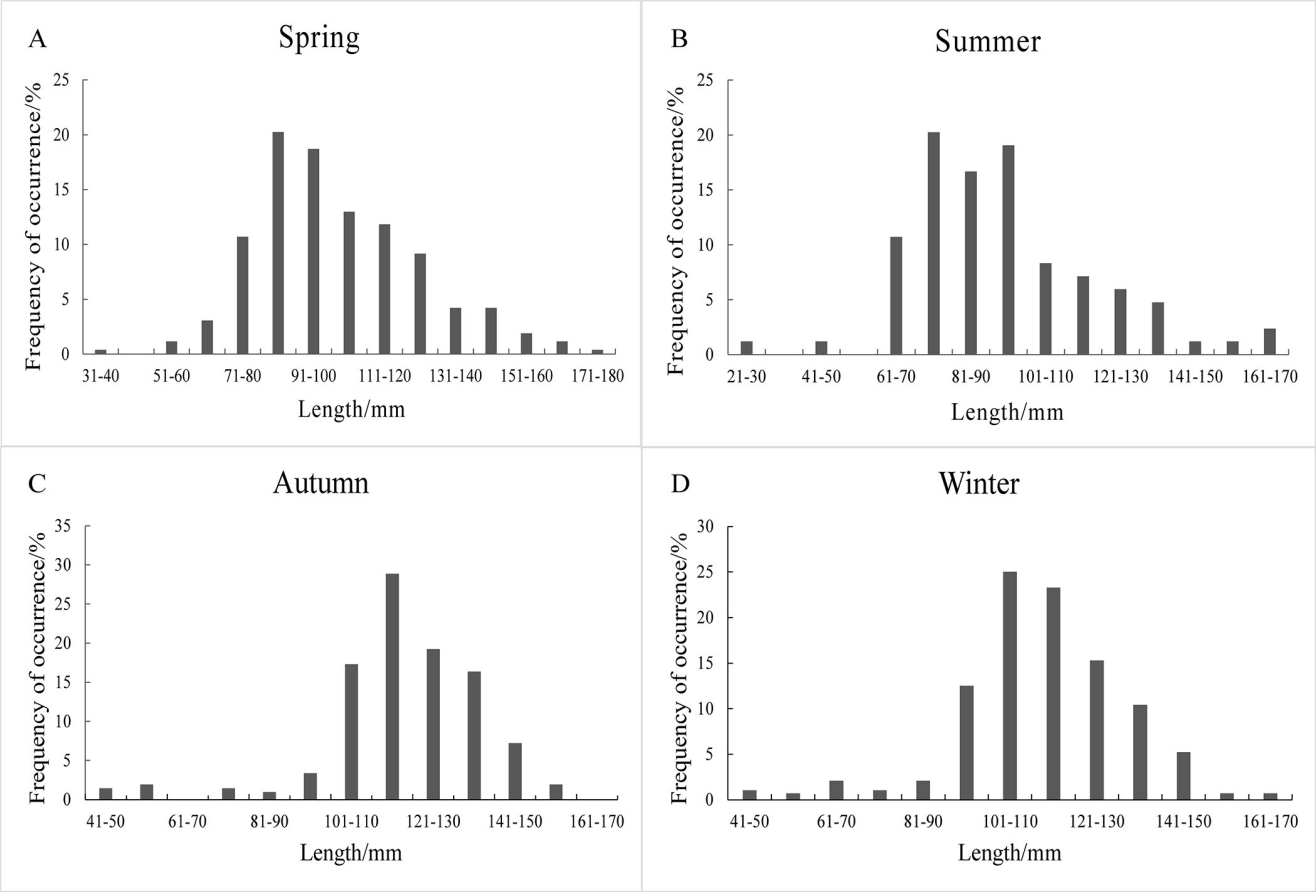
(A)The length distribution of *J*.*belangerii* in spring 2020. (B)The length distribution of *J*.*belangerii* in summer 2020. (C)The length distribution of *J*.*belangerii* in autumn 2020. (D)The length distribution of *J*.*belangerii* in winter 2020.

Examining the distribution of length and weight among the 842 samples of *Johnius belangerii* in Zhanjiang Bay throughout 2020 revealed distinct seasonal variations. Specifically, in spring, the length of samples ranged from 34 to 174 mm, with an average length of 102 mm. The dominant length range was 71 to 120 mm, accounting for 74.43% of the samples length. The associated weight spectrum spanned 0.95 to 125.22 g, with an average of 26.30 g and a dominant weight category of 5 to 20 g. In summer, specimens ranged from 27 to 162 mm in length exhibited an average of 94 mm. The dominant length range was 61 to 100 mm, representing 66.67% of the samples. The weight ranged from 0.33 to 105.12 g, with an average of 21.08 g and a dominant weight span of 0 to 25 g, accounting for 77.38% of the samples. In autumn, specimens ranging from 42 to 160 mm presented an average length of 118 mm, primarily clustered between 101 to 140 mm (81.73%). The corresponding weight range was 1.00 to 83.91 g, with an average weight of 34.95 g, predominantly concentrated between 20 to 40g (56.73%). Winter samples, ranging from 48 to 167mm in length, exhibited an average length of 113mm with a dominant length between 91 to 140mm (86.46%). The weight range was 1.17 to 107.40 g, with an average weight of 31.82g and a dominant weight of 15 to 35 g (59.72%) (Fig 4). Overall, seasonal variations significantly influenced the dominant length and weight distribution of *Johnius belangerii* across the year.

### 3.2. Relationship between length and weight

**Fig 5:**
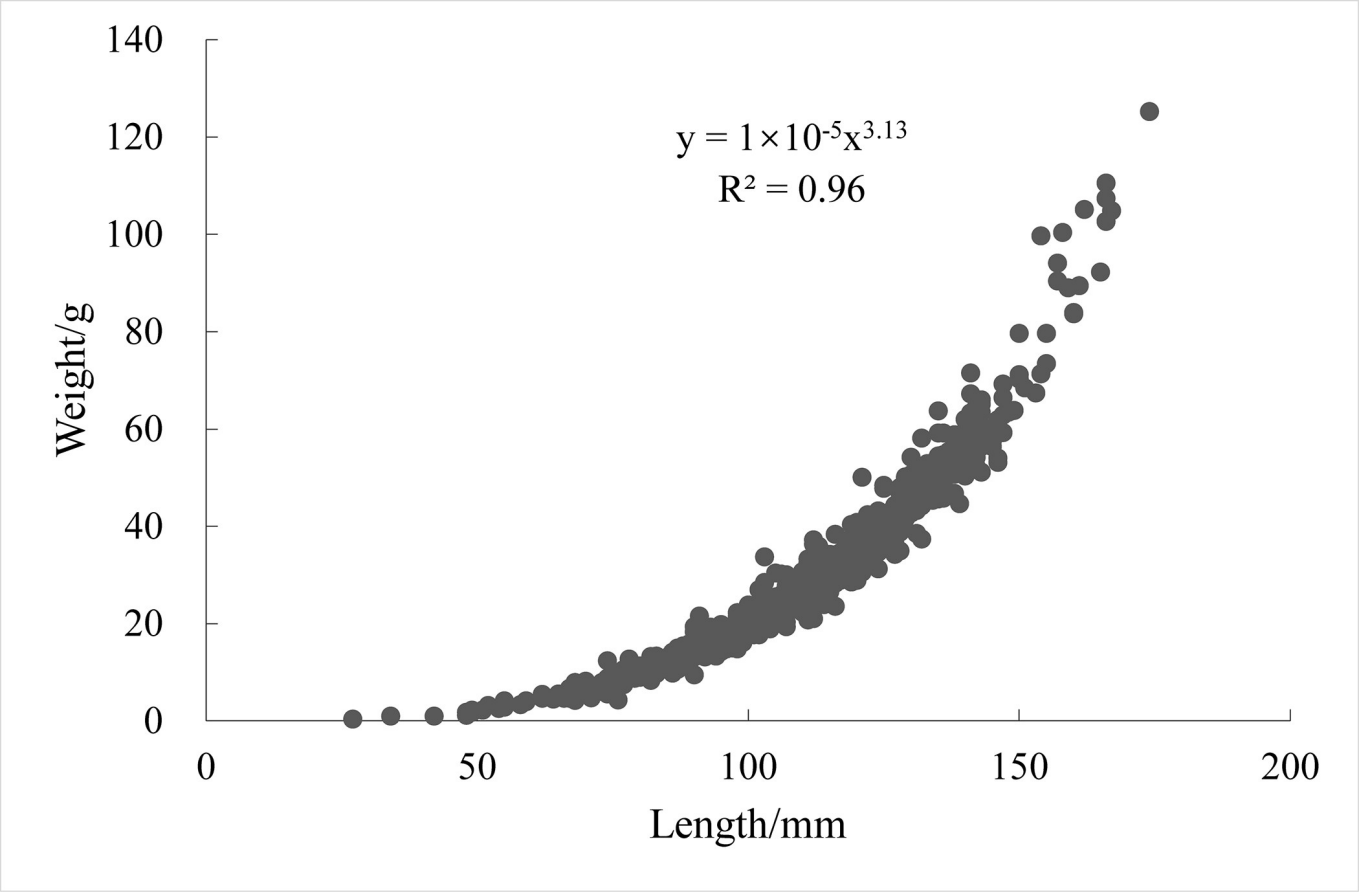
Relationship between length and weight of *J*.*belangerii* in 2020.

The relationship between length and weight of *Johnius belangerii* in Zhanjiang Bay was modeled using a power function (Fig 5). The expression for the relationship between length and weight in 2020 was *W* = 10^-5^*L*^3.13^, (R^2^ = 0.96, n = 842). The condition factor for growth was 1 × 10^−5^, and the allometric growth factor *b* was 3.13, indicating positive allometric growth. Statistical analysis of length and weight parameters across different periods and genders revealed consistent positive allometric growth for all individuals (Table 2).

**Table 2:** Relation parameters between length and weight of *J*.*belangerii*.

Statistical category	a	b	R^2^	N
Spring	1×10^−5^	3.16	0.97	262
Summer	9×10^−6^	3.18	0.97	84
Autumn	1×10^−5^	3.12	0.95	208
Winter	9×10^−6^	3.16	0.97	288
Males	1×10^−5^	3.09	0.96	255
Females	1×10^−5^	3.15	0.97	193
Pooled	1×10^−5^	3.13	0.96	842

### 3.3. Relationship of length to sex ratio and gonadal maturity

**Fig 6:**
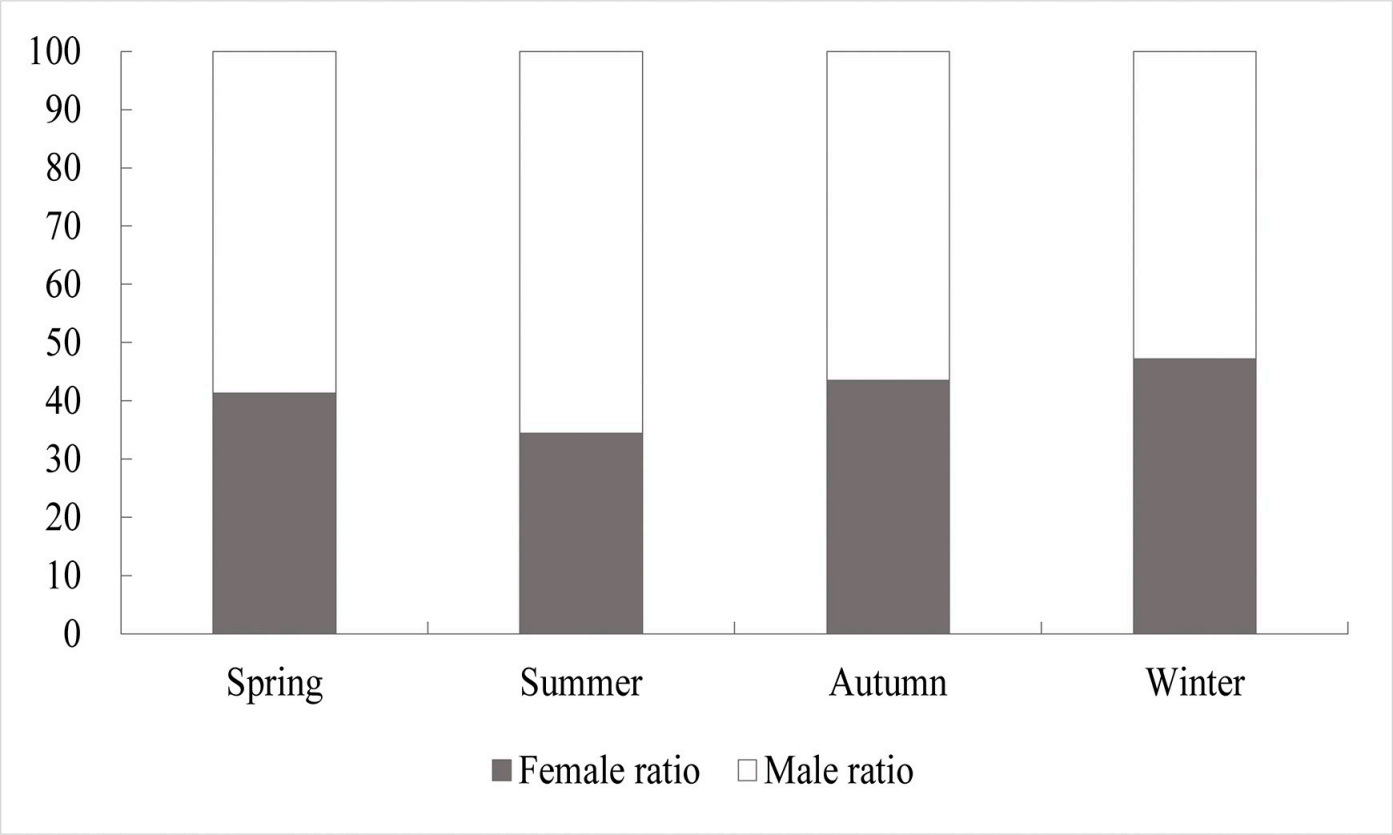
Sex ratio by season for *J*.*belangerii*.

**Fig 7:**
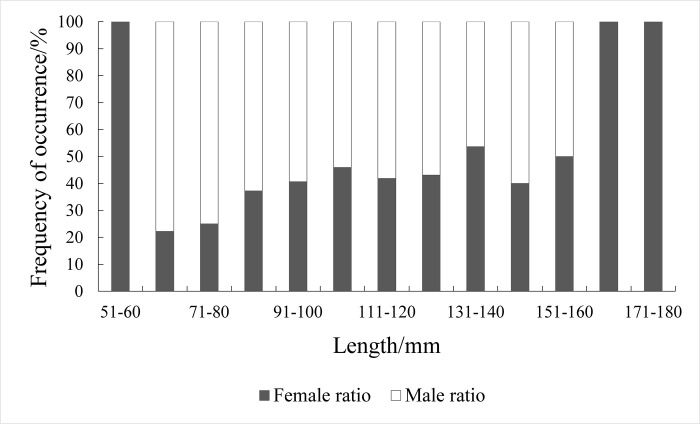
Sex ratio of different size group for *J*.*belangeri*.*i*.

Among the individuals with identifiable sex, 193 were females and 255 were males, resulting in a sex ratio of 1:1.32 An additional 52 individuals at Stage I could not be sexed. The sex ratio v exhibited minimal seasonal variation, with male-to-female ratios of 1.42:1, 1.90:1, 1.3:1 and 1.12:1, across the respective seasons (Fig 6). The chi-square test indicated no significant deviation from the expected 1:1 sex ratio for *Johnius belangerii* (χ^2^ = 0.041, P>0.05), nor were there significant seasonal differences (χ^2^ = 2.835, P>0.05) (Fig 7). The number of males and females showed a parabolic distribution with length. Males predominated in the <61 mm and ≥161 mm length groups, while females were more evenly distributed across all lengths. Overall, the number of males in this collection was high and concentrated, while the length of female phases was relatively more widely distributed.

**Fig 8:**
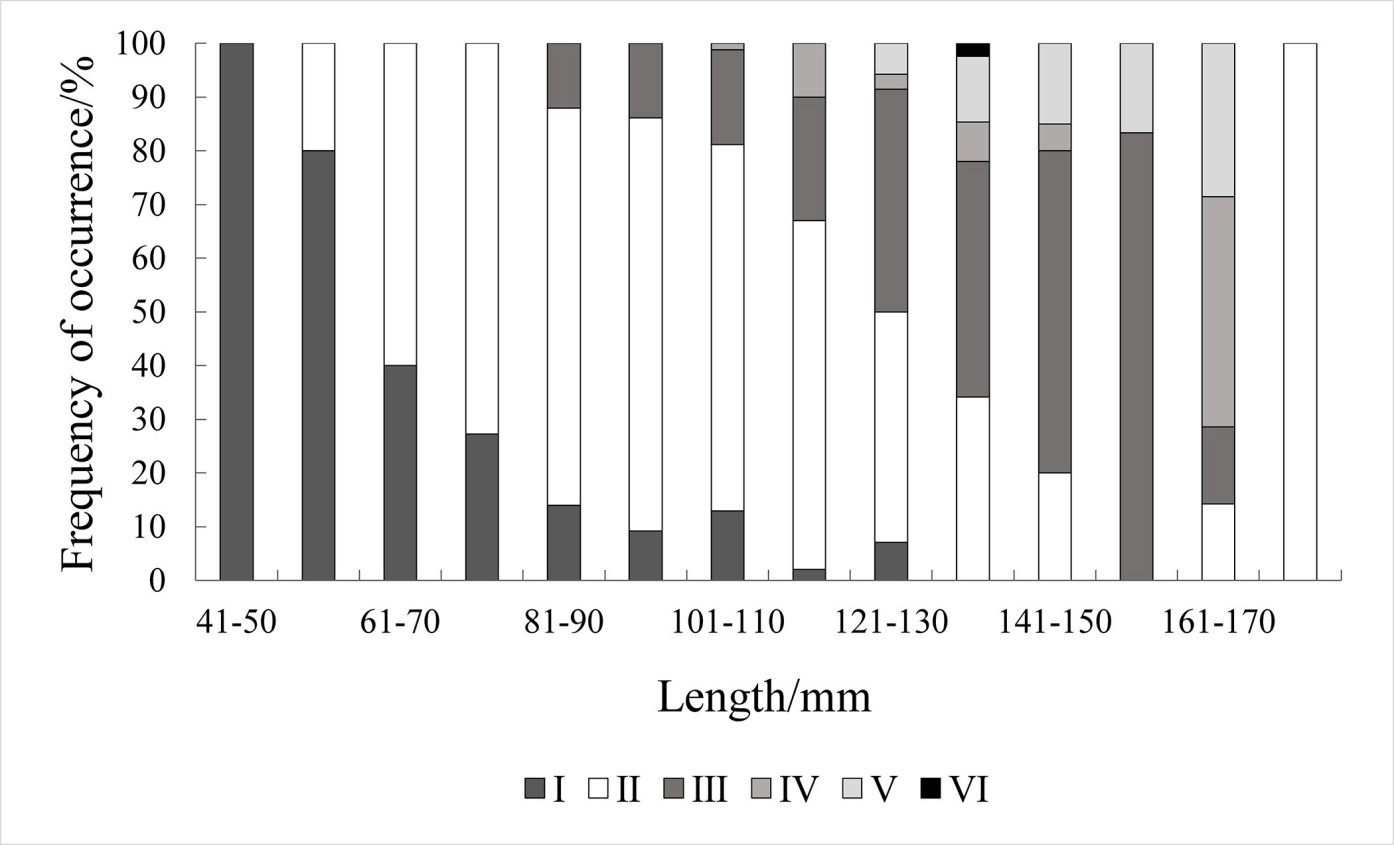
Maturity stage varies with size for *J*.*belangerii*.

Gonadal maturity analysis of 500 *Johnius belangerii* indicated that individual’s gonadal maturity at Stage I (10.4%) and Stage II (58.8%) were predominant, followed by Stage III (23.6%), Stage IV (4.0%), Stage V (3.0%), and Stage Ⅵ (0.2%). Sexually mature individuals (Stage III and above) totalled 154 (30.8%), primarily within the length 101–150 mm range (82.5%). Gonadal maturity in *Johnius belangerii* was predominantly stage I when the length was <61mm; stage II was predominant in the range of 61–130 mm; except for 161–180 mm, gonadal maturity in *Johnius belangerii* was predominantly stage III when the length was ≥131 mm. Sexually mature individuals began to appear when length was ≥81 mm (Fig 8). *Johnius belangerii* typically has a lifespan of 3 to 5 years and is a seasonal breeding species, with its reproductive period generally occurring in spring and summer [21,31–34]. In this study, sexually mature individuals ranged in length from 82–174 mm, and the sampling was conducted in January, April, August, October, November and December (data from January were insufficient for further analysis). A higher number of sexually mature individuals were observed in April and August, consistent with the species’ known life history characteristics.

### 3.4. Estimation results of life history parameters

**Fig 9:**
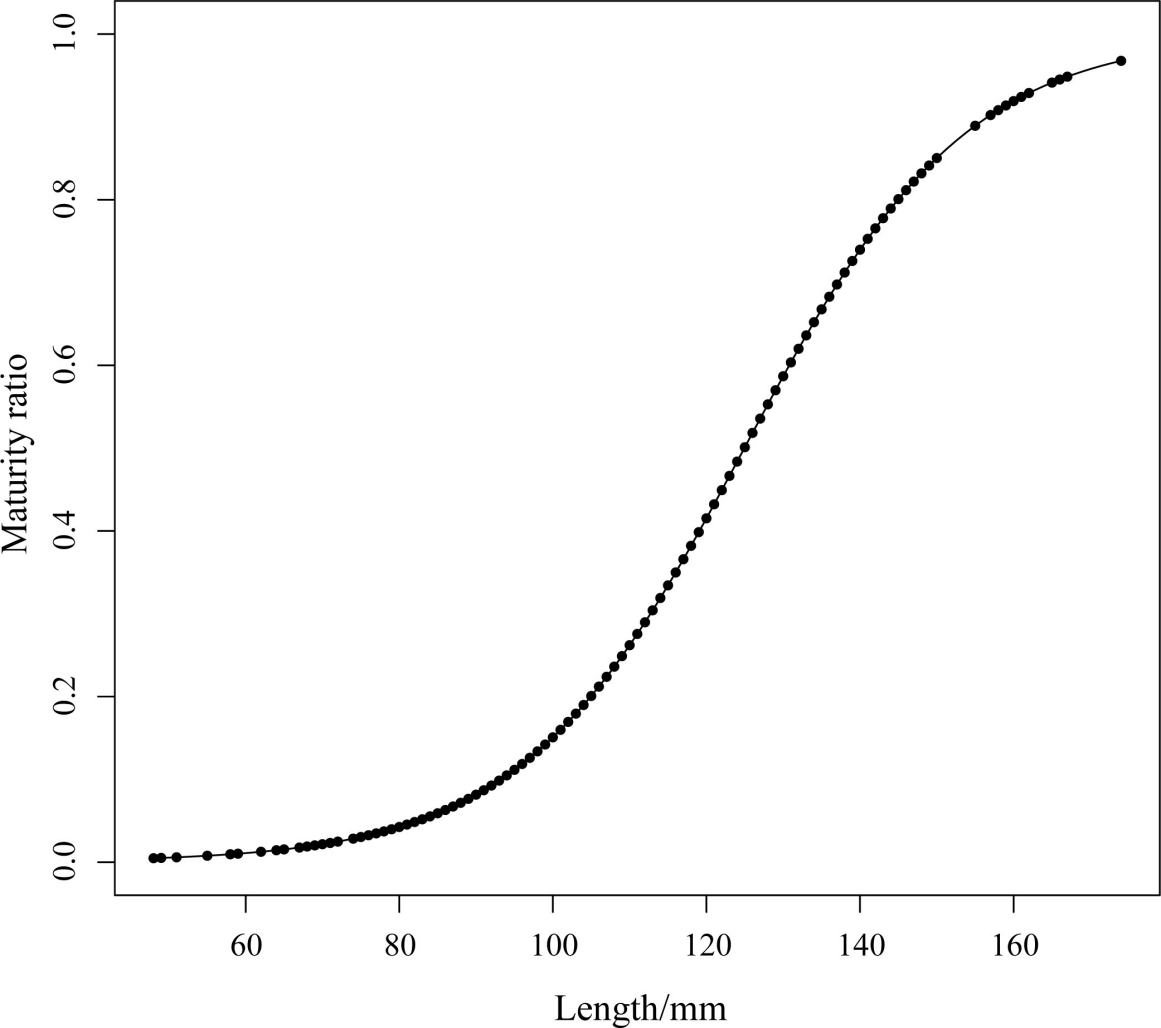
The fitted relationship between length and the percentage of mature of *J*. *belangerii*.

The range of sexually mature length in the 2020 catch population of *Johnius belangerii* was 82 to 166 mm, with an average length of 125 mm. The fitted curve indicated a first sexual maturity length (*L*_50_) of 125 mm, and a 95% sexual maturity length(*L*_95_) of 167 mm (Fig 9). Minimum sexually mature lengths were 91mm for females and 82 mm for males.

The estimated asymptotic length *L*_*∞*_ was 180 mm, with a growth coefficient (*k*) of 0.58, total mortality (*Z*) of 2.00, natural mortality (*M*) of 0.73, and fishing mortality (*F*) of 1.27. The *M*/*k* ratio was 1.26, the *F*/*M* ratio was 1.74, and the exploitation rate (*E*) was 0.64, indicating overfishing. The *Z*/*k* value of 3.45 further confirmed that overfishing significantly impacted *Johnius belangerii* mortality.

### 3.5. Population parameters and potential reproduction rate of *Johnius belangerii*

**Fig 10:**
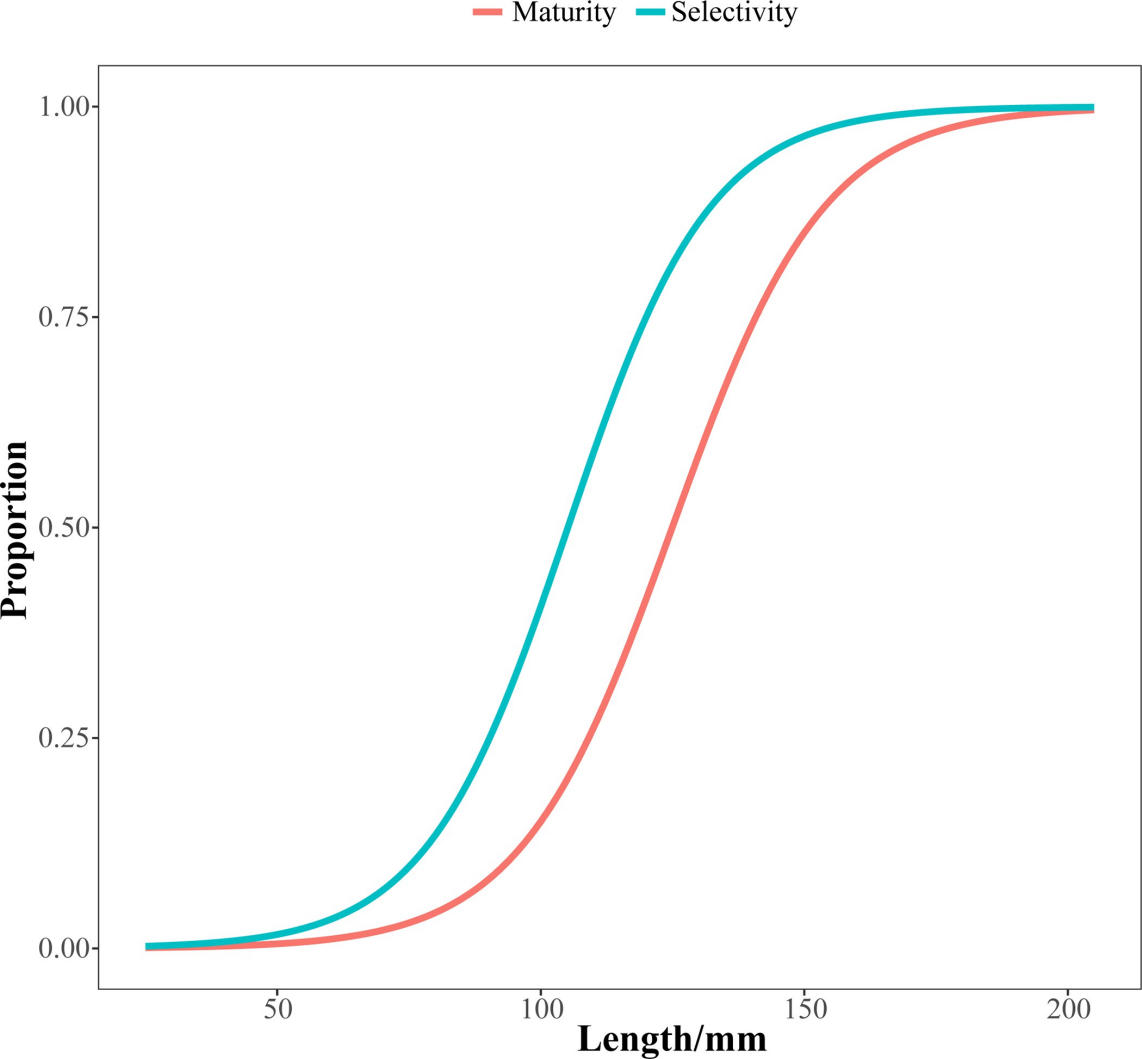
Selectivity curves and maturity curve of *J*.*belangerii* in Zhanjiang Bay.

**Fig 11:**
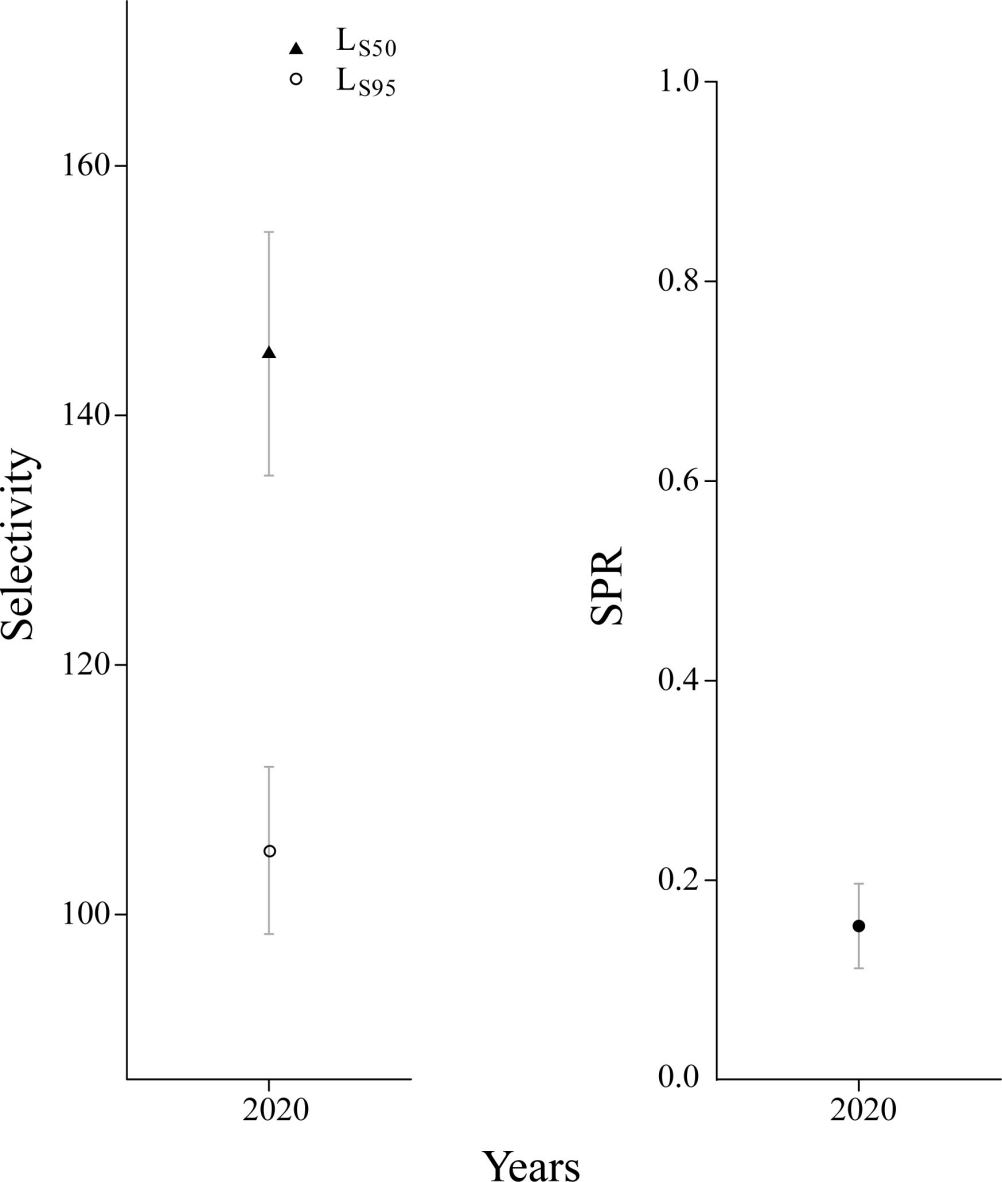
Expected length of sexual maturity and SPR.

The maturity curves at the same length were higher than expected, suggesting earlier sexual maturity for *Johnius belangerii* (Fig 10). The model results show that the mean values of *L*_S50_ and *L*_S95_ were 105 mm and 145 mm, which were notably smaller than initial sexually mature lengths (*L*_50_) and 95% sexually mature lengths (*L*_95_). And the SPR value was only estimated to be 0.15 (Fig 11), indicating significant reproductive challenges for the population.

## 4 Discussion

This study represents the first comprehensive analysis of the growth characteristics and community composition of *Johnius belangerii* in Zhanjiang Bay. It identifies key differences in the *b*-value and dominant length groups, and explores the reasons behind these variations. The reproductive potential ratio of *Johnius belangerii* was estimated using the LBSPR method. The results were validated by comparing them with traditional exploitation rate calculations, indicating a trend of miniaturization and low reproductive potential in the population. Based on these findings, conservation strategies for *Johnius belangerii* are proposed, and limitations of the LBSPR model regarding input values are discussed.

Global studies on the relationship between length and weight of a marine fishes have established that the *b*-value for most marine fish typically ranges between 2.5 and 3.5, with *b*-values close to 3 indicating isometric growth [35]. In this study, the *b*-value of *Johnius belangerii* was 3.13, higher than the values reported in the Yellow Sea and Bohai Sea (2.5745) [35], the northern South China Sea (2.653) [36], as well as the East China Sea (3.046) [37] and the Pearl River Estuary waters (2.876) [32]. These differences reflected the variations in the nutrient conditions and environmental factors, as well as life stage differences across regions. Notably, the *b*-value of *Johnius belangerii* in Zhanjiang Bay was lowest in autumn. This may presumably due to the breeding season of *Johnius belangerii* in July-August [21] and the rapid juvenile growth leads to a lower growth parameter *b*-value, which requires further confirmation. The lengths of *Johnius belangerii* in Zhanjiang Bay ranged from 27 to 174 mm, with the dominant length group ranging from 91 to 130 mm. Compared to other areas, the range of lengths of *Johnius belangerii* in this area was larger and the dominant length group values differed, primarily due to varying fishing activities in different seas (Table 3).

**Table 3:** Range of length and weight of *J*.*belangerii* in different sea areas.

area	BLR/BLM (mm)	ref
Haizhou Bay	47~138	Zhang Yunlei et al. 2020 [38]
	92~106	
Ma an Archipelago sea	30~264	Wang K et al. 2012 [21]
	80~100; 130~180	
the South of Zhejiang	43~202	Pei Rude et al.2021 [39]
	99.9~172.8	
the Pearl River Estuary	18~167	Li Y Z et al.2014 [32]
Xiamen Bay	5~201	Shen S C et al.2020 [33]
	80~120	
Liusha Gulf sea area	80~191	Hui G et al.2017 [34]
	121–140	
this paper	27–174	
	91~130	

Notes: BLR/BLM is the length range/dominant length group.

The uncertainty in life history parameter estimates obtained from different methods were found to be within acceptable limits (Table 4). Consequently, the estimates from ELEFAN I method within FISAT II software were selected for the overall results. This study also consulted data from relevant published literature, confirming that the high exploitation rate of *Johnius belangerii* is not an isolated case. Overall, the estimated parameters demonstrated high accuracy, ensuring the reliability of the subsequent SPR. The stock status of *Johnius belangerii* in Zhanjiang Bay is concerning. Despite being a dominant species with considerable resource availability, its exploitation status remains uncertain. The estimated exploitation rate of *Johnius belangerii* in Zhanjiang Bay was 0.64. According to Gulland [40], the optimum exploitation is around 0.5, indicating over-exploited of *Johnius belangerii* in Zhanjiang Bay. Additionally, the LBSPR model estimated an SPR of 0.15, below the internationally accepted threshold of 20%. Both the high exploitation rate and the low SPR suggest that intensive fishing and altered trophic conditions are advancing the age of sexual maturity as a self-regulatory mechanism [41]. The small actual sexually mature lengths length (*L*_S50_ and *L*_S95_) suggests that a significant proportion of the *Johnius belangerii* population in Zhanjiang Bay is being harvested before spawning, exacerbating resource depletion and hindering sustainable development. This miniaturization and early sexual maturity response to fishing pressure deteriorates population structure and jeopardizes fishery sustainability.

**Table 4:** Estimation and comparison of life history parameters.

	FISAT Ⅱ	TropicFishR	Reference 1 [31]	Reference 2 [33]
L_∞_(mm)	179.55	185.39	185.85	220.50
K	0.58	0.60	0.61	0.56
Z	2.00	2.66	2.40	2.25
M	0.73	0.74	0.75	0.63
F	1.27	1.92	1.65	1.62
E	0.64	0.72	0.69	0.72
M/K	1.26	1.23	1.23	1.13
F/M	1.74	2.59	2.20	2.57
Z/K	3.45	4.43	3.93	4.02

While the LBSPR method does not provide specific exploitation rate, it simplifies the assessment process by avoiding tedious calculations for each life-history parameter. It effectively clarifies the current stock status of *Johnius belangerii* confirming its utility in data-limited fisheries resource assessments and offering a streamlined approach to measure the impact of fishing on potential productivity in the northern Zhanjiang Bay South China Sea.

To ensure the sustainable development of *Johnius belangerii* in Zhanjiang Bay appropriate conservation strategies are necessary. Jeremy Princ et al [42]. recommend setting the minimum allowable size for fishing to 1.1 to 1.2 times the length of the first sexual maturity (*L*_50_), translating to 137 to 150 mm. This implies the dual objectives conserving the stock and securing a substantial yield even under elevated fishing pressure. Beyond extending the duration of the fishing season, it is imperative to implement and reinforce fisheries management practices. Long-term tracking and monitoring of resources are not only fundamental for fishery stock assessment but also serve as critical indicators of changes in fishery resources and the ecosystem services provided by the respective waters. Therefore, it is particularly crucial to focus on the tracking and monitoring of fishery resources and their ecological environment in Zhanjiang Bay, to obtain prolonged Zhanjiang Bay and continuous monitoring data [43], This approach can mitigate damage to the marine ecosystem in a timely manner, thereby ensuring the sustainable use of *Johnius belangerii* in Zhanjiang Bay.

Although Chong et al. [18] stated that LBSPR is one of the most accurate length-based assessment method, Hordyk et al. [14] demonstrated that the LBSPR model exhibits significant sensitivity, primarily due to the high demands on the model input values [13]. The accuracy of the *L*_*∞*_ parameter is crucial for the fitting results, as its misestimation can lead to substantial discrepancies. Additionally, the parameters *M*/*k*, *L*_50_ and *L*_95_ also influence the results, albeit to a lesser extent. Scientific assessments are vital for fisheries management and decision-making.

In the context of data limitations affecting most of China’s offshore fisheries, this study analyzed the biological characteristics and community composition of *Johnius belangerii* in Zhanjiang Bay in 2020. The reproductive potential ratio of the species was estimated using the LBSPR model, the reliability of this method was evaluated. The findings revealed evidence of individual miniaturization and reduced reproductive potential in *Johnius belangerii* within Zhanjiang Bay. Based on the results, the study suggests the development of a resource conservation and management strategy for *Johnius belangerii* in Zhanjiang Bay.

The study also of the study highlighted the limitations of the LBSPR model, particularly with respect to input values. To improve the model’s accuracy, future research should focus on further development and refinement of the approach using length data, as well as validating its applicability for other small-scale fisheries stock assessments. For instance, incorporating a more complex model structure that integrates environmental factors and biological parameters would allow for more comprehensive investigations and assessments of fishery resources.

## Supporting information

S1 File (XLSX)
