# Correction: Assessing the exploitation status of *Johnius belangerii* in Zhanjiang Bay

**DOI:** 10.1371/journal.pone.0316272

**Published:** 2024-12-19

**Authors:** Siman Deng, Dongrong Liao, Kun Lin, Shaoliang Lyu, Ning Chen, Xuefeng Wang

The Funding statement for this paper is incorrect. The correct Funding statement is as follows: This study is supported by the Sino-Indonesian technical cooperation in coastal marine ranching, Asian Cooperation Fund Program (12500101200021002), the program for scientific research start-up funds of Guangdong Ocean University (060302022301), Asian Cooperation Fund Program on Modern Fishery Cooperation between China and Other Coastal States of the South China Sea (102125241620040000001), the College Students’ Innovation and Entrepreneurship Project of Guangdong Province (S202210566011), and the College Student Innovation Team Project of Guangdong Ocean University (CCTD201803). The funders have no role in study design, data collection and analysis, decision to publish, or preparation of the manuscript.
